# Comprehensive analysis of complications after transperineal prostate biopsy without antibiotic prophylaxis: results of a multicenter trial with 30 days’ follow-up

**DOI:** 10.1038/s41391-021-00423-3

**Published:** 2021-07-15

**Authors:** Tobias Kohl, August Sigle, Timur Kuru, Johannes Salem, Hanjo Rolfs, Tobias Kowalke, Rodrigo Suarez-Ibarrola, Jakob Michaelis, Nadine Binder, Cordula A. Jilg, Arkadiusz Miernik, Markus T. Grabbert, W. Schultze-Seemann, Christian Gratzke, Daniel Porres

**Affiliations:** 1 Department of Urology, Klinikum Leverkusen, Leverkusen, Germany; 2 Department of Urology, Faculty of Medicine, Medical Centre – University of Freiburg, Freiburg, Germany; 3 Praxis am Ebertplatz, Cologne, Germany; 4 Urology Department, Clinic LINKS VOM RHEIN, Cologne, Germany; 5 Institute of Digitalization in Medicine, Faculty of Medicine, Medical Centre – University of Freiburg, Freiburg, Germany

**Keywords:** Cancer therapy, Cancer screening

## Abstract

**Background:**

To investigate infectious and non-infectious complications after transperineal prostate biopsy (TPB) without antibiotic prophylaxis in a multicenter cohort. Secondly, to identify whether increasing the number of cores was predictive for the occurrence of complications. Thirdly, to examine the relation between TPB and erectile dysfunction.

**Methods:**

We analyzed a retrospective multicenter cohort of 550 patients from three different urological centers undergoing TPB without antibiotic prophylaxis. The median number of cores was 26. Demographic and clinical data were extracted by reviewing patients’ electronic medical records and follow-up data such as postoperative complications obtained by structured phone interviews. To investigate the influence of the number of cores taken on the occurrence of complications, we performed univariate and multivariate mixed effects logistic regression models.

**Results:**

There was no case of sepsis reported. Overall, 6.0% of patients (33/550) presented with any complication besides mild macrohematuria. In all, 46/47 (98%) complications were ≤Grade 2 according to Clavien–Dindo. In multivariate regression analyses, an increased number of cores was associated with overall complications (odds ratio (OR) 1.08, 95% confidence interval (CI) 1.02–1.14, *P* = 0.01) and specifically bleeding complications (OR 1.28, 95% CI 1.11–1.50, *P* = 0.01) but not with infectious complications (OR 1.03, 95% CI 0.97–1.10, *P* = 0.67). A total of 14.4% of patients referred impairment of erectile function after TPB. Of note, 98% of these men were diagnosed with prostate cancer.

**Conclusions:**

This is the first multicenter trial to investigate complications after TPB without antibiotic prophylaxis. In our study, we found no case of sepsis. This underlines the safety advantage of TPB even without antibiotic prophylaxis and supports the ongoing initiative to abandon TRB of the prostate. A higher number of cores were associated with an increase in overall complications specifically bleeding complications, but not with infectious complications. Post-biopsy erectile dysfunction was mainly present in patients diagnosed with PCa.

## Introduction

Until recently, transrectal biopsy (TRB) has been the real-world standard for the histopathological diagnosis of prostate cancer (PCa) [1]. Despite antibiotic prophylaxis, a dramatic rise in hospital readmission rates of up to 10% and even in 30 days’ mortality has been reported in recent years [2, 3]. Fortunately, transperineal prostate biopsy (TPB) offers a promising solution to this current challenge. In a recent meta-analysis including seven studies comparing TRB to TPB, the transperineal approach significantly decreased the risk of complications such as urogenital infections, rectal bleeding, and fever [4]. Moreover, TPB has shown an improved diagnostic sensitivity for clinically significant PCa [5]. Considering TPB as a sterile procedure, restricting the use of antibiotics would be beneficial to address the global antibiotic resistance crisis and to avoid medication side effects on an individual level [6, 7]. The primary aim of the study was to investigate the incidence of infectious and non-infectious complications after TPB without antibiotic prophylaxis in a multi-institutional setting. Second, we aimed for quantifying a potential association of number of cores taken on the subsequent occurrence of complications. Moreover, we analyzed whether there was any correlation between the number of cores taken and the occurrence of complications. The third objective was to examine the relation between TPB and erectile dysfunction.

## Patients and methods

### Study population

We retrospectively obtained data from a multicenter cohort of 605 consecutive patients who underwent TPB in one of the three participating medical centers. As this study was planned as a pilot study, the sample size was chosen with regard to the effort of data collection and acquisition periods were limited to 1 year. University Hospital Freiburg (UHF), Freiburg, Germany, Municipal Hospital Leverkusen (MHL), Leverkusen, Germany, and one urological practice (UP) located in Cologne, Germany. Acquisition periods varied slightly between centers: UHF January 2019–December 2019, MHL August 2019–July 2020, and UP March 2019–July 2020. The indication for TPB was based on elevated prostate-specific antigen (PSA), abnormal digital rectal examination, suspicious findings in multiparametric MRI (mpMRI), or as part of the re-biopsy routine under active surveillance. Fifty-five patients were excluded due to peri-interventional antibiotic therapy. Five hundred fifty men were included for final analyses. Institutional ethics approval was obtained (288/16). Due to the retrospective nature and minimal risk of the study, written informed consent was waived. This study was performed in accordance with the Declaration of Helsinki.

### Biopsy procedure

MpMRI–TRUS fusion prostate biopsy was performed using either a robotic-assisted approach with iSRobot Mona LisaTM^®^ (Biobot Surgical, Singapore) at UHF or using the BiopSee^®^ system (MedCom, Darmstadt, Germany) with a template guided approach at MHL and at the urologic practice. Procedural details have been previously described in Refs [8] and [9], respectively. All centers applied a combined biopsy strategy by targeting suspicious lesions and performing a simultaneous systematic biopsy. Depending on target size, at least two cores were taken per target. Systematic biopsy was performed according to the Ginsburg scheme [10]. The number of systematic cores was dependent on prostate size. A median of 26 cores was taken per individual. The number of biopsy cores in the final analysis refers to the total number of cores (target cores plus systematic cores). The procedure was performed in lithotomy position under general anesthesia. Preoperative perineal preparation included an antiseptic wash with Octenidindihydrochloride/Phenoxyethanol or Povidon-Iod. Antibiotic prophylaxis was not administered. Alpha-blockers were not administered routinely as peri-interventional treatment.

### Data collection and statistical analysis

Demographic and clinical data were extracted by reviewing patients’ electronic medical records. Baseline characteristics included age, prostate volume by MRI, PSA, number of biopsy cores, history of previous biopsy, mpMRI findings according to the Prostate Imaging: Reporting and Data System (PI-RADS) and histopathological prostate biopsy findings according to the classification of the International Society of Urological Pathology (ISUP). For follow-up, data such as postoperative complications from patients’ electronic medical records were validated and complemented by structured phone interviews. Follow-up data included infectious complications such as urinary tract infections (UTI), prostatitis and urosepsis, bleeding complications, acute urinary retention (AUR), and erectile dysfunction. UTI was defined as bacteriuria with concomitant clinical symptoms such as dysuria, urinary frequency, or suprapubic pain. Erectile function impairment was classified dichotomously as worsened or unchanged. Complications were reported according to the Clavien–Dindo classification (CD) [11]. AUR and macrohematuria treated with catheter insertion and irrigation were considered Clavien–Dindo grade 1. Continuous variables are described as mean with standard deviation (SD). Categorical variables are described with absolute counts and percentages. To investigate the influence of the number of cores taken on the occurrence of complications, we used univariate and multivariate mixed effects logistic regression models. Models incorporated the covariates age, prostate volume, PSA, number of cores, history of previous biopsy, active surveillance status, and ISUP category as fixed effects, with center as random intercept. Stepwise backward elimination with Akaike information criterion as stopping criterion was used to select an important subset of covariates. *P* value <0.05 was considered statistically significant. SPSS^©^ statistics 27 (IBM, Armonk, New York, USA) and R version 4.0.3 [12] were used for statistical analyses.

## Results

Table 1 presents the baseline characteristics of the study cohort. The mean age and PSA were 68.3 years (SD 8.1) and 9.8 ng/ml (SD 9.6), respectively. The mean number of biopsy cores taken was 25.4 (SD 7.6) and 183 patients (33.3%) had a history of previous biopsy. The overall cancer detection rate was 64.9% and clinically significant cancer defined as ISUP > 2 was diagnosed in 259 patients (47.0%). Follow-up data on complications could be retrieved for all patients, except for erectile function (*n* = 348, 63.3%). Complications are shown in Table 2. The total proportion of patients with any complication besides mild, self-limiting macrohematuria was 6.0% (33/550). Forty-six out of 47 (98%) complications were ≤CD Grade 2. There was a single case of hemorrhoidal bleeding that was repaired endoscopically (CD 3a) with no subsequent consequences. Infectious complications were reported in 20 men (3.6%) without there being a single case of sepsis. Five patients developed UTI with fever (0.9%) and were treated with a course of antibiotics. Only one out of five men required hospital readmission due to UTI with fever that warranted intravenous antibiotic therapy. Relevant bleeding complications requiring irrigation and/or the evacuation of a bladder tamponade by flushing via the indwelling catheter occurred in seven and two men, respectively (in sum 1.5%). AUR was reported in 11 cases (2.0%) that were all resolved by temporal catheterization. Data for erectile function were available in 348 cases whereby 50 men (14.4%) stated an impaired function. Of note, 98% of these patients were diagnosed with PCa.

**Table 1:** Baseline characteristics from 550 patients.

Parameter	Value – Mean (+SD)
*Age (years)*	68.3 (±8.1)
*Prostate volume (ccm)*	58.3 (±30.4)
*PSA (ng/ml)*	9.8 (±9.6)
*Number of cores*	25.4 (±7.6)
*History of previous biopsy, n (%)*	183 (33.3)
*PI-RADS*	*n*/*n* (%)
2	15/550 (2.7)
3	84/550 (15.3)
4	266/550 (48.4)
5	143/550 (26.0)
n/a	42/550 (7.6)
*Cancer detection rate*	*n*/*n* (%)
Any cancer	357/550 (64.9)
ISUP 1	98/550 (17.1)
≥ISUP 2	259/550 (47.0)
≥ISUP 3	115/550 (20.9)

*SD* standard deviation, *PSA* prostate-specific antigen, *PI-RADS* Prostate Imaging: Reporting and Data System, *ISUP* International Society of Urological Pathology.

**Table 2:** Complications after transperineal biopsy in 550 patients.

Parameter	Value – *n*/*N* (%)
*Any complication*^a^	33/550 (6.0)
Complications according to Clavien–Dindo (CD)	
1	20/550 (3.6)
2	26/550 (4.7)
3a	1/550 (0.2)
*Any infectious complication*^b^	20/550 (3.6)
UTI with need of antibiotic therapy (CD 2)	19/550 (3.5)
UTI with fever >38.5 °C (CD 2)	5/550 (0.9)
Prostatitis (CD 2)	2/550 (0.4)
Urosepsis or ICU (CD 4)	0/550 (0)
Death within 30 days (CD 5)	0/550 (0)
*Any relevant bleeding complication*^c^	8/550 (1.5)
Macrohematuria with irrigation (CD 1)	7/550 (1.3)
Bladder tamponade (CD 1)	2/550 (0.4)
Macrohematuria with TUR intervention (CD 3)	0/550 (0)
Mild macrohematuria (no CD)	212/550 (38.5)
Acute urinary retention (CD 1)	11/550 (2.0)
Erectile dysfunction (no CD)	50/348 (14.4)

*UTI* urinary tract infection, *ICU* intensive care unit, *TUR* transurethral.

^a^Total number of patients with one or more complications besides mild macrohematuria.

^b^Total number of patients with any infectious complication.

^c^Total number of patients with bleeding complications besides mild macrohematuria.

Table 3 shows the results of the mixed effects logistic regression models for the prediction of complications. To investigate whether the number of cores was associated with complications, we calculated univariate and multivariate analyses. In the multivariate model, a higher number of cores was significantly associated with the occurrence of any complication (OR 1.08, 95% CI 1.02–1.14, *P* = 0.01), specifically bleeding complications as specified in Table 2 (OR 1.28, 95% CI 1.11–1.50, *P* = 0.001) and AUR (OR 1.13 95% CI 1.03–1.26, *P* = 0.02). Higher age was also a significant predictor for bleeding complications (OR 1.15, 95% CI 1.03–1.31, *P* = 0.02). Neither the number of biopsy cores (OR 1.03, 95% CI 0.97–1.11, *P* = 0.35) nor any of the other examined parameters had a significant influence on the occurrence of infectious complications such as UTI, UTI with fever, prostatitis, or urosepsis. In univariate analysis, larger prostate volumes were associated with more overall complications (OR 1.02, 95% CI 1.00–1.02, *P* = 0.01) and increased the likelihood for developing post-procedural AUR (OR 1.02, 95% CI 1.00–1.03, *P* = 0.01). These findings were not confirmed in multivariate analyses.

**Table 3:** Results of univariate and multivariate mixed effects logistic regression analyses.

	Univariate		Multivariate	
	OR (95% CI)	*P*	OR (95% CI)	*P*
*Prediction of any complication*				
Number of cores (*n*)	1.08 (1.02–1.15)	0.01**	1.08 (1.02–1.14)	0.01**
Prostate volume (ccm)	1.02 (1.00–1.02)	0.01**	Not significant	
*Prediction of any infectious complication*				
Number of cores (*n*)	1.04 (0.97–1.12)	0.31	1.03 (0.97–1.10)^a^	0.67^a^
*Prediction of any relevant bleeding complication*				
Number of cores (*n*)	1.24 (1.09–1.42)	<0.01**	1.28 (1.11–1.50)	<0.01**
Age (years)	1.13 (1.02–1.27)	0.03*	1.15 (1.03–1.31)	0.02*
*Prediction of acute urinary retention*				
Number of cores (*n*)	1.13 (1.03–1.26)	0.02*	1.13 (1.03–1.26)	0.02*
Prostate volume (ccm)	1.02 (1.00–1.03)	0.01**	Not significant	

Multivariate analyses was conducted with incorporation of the covariates number of cores, age, prostate volume, PSA, history of previous biopsy, active surveillance status, and ISUP category. Besides for the covariate number of cores, only significant results are shown.

*OR* odds ratio, *CI* confidence interval.

Asterisks indicate statistical significance: *P < 0.05; **P < 0.01.

^a^Results from full multivariate model, i.e., w/o model selection.

## Discussion

An alarming increase of potentially life-threatening infectious complications has been reported after TRB despite the standard use of antibiotic prophylaxis [2, 3]. Moreover, there is a global need to restrict antibiotic use due to a dramatic rise in resistance rates [6]. Due to the sterility of the procedure and punctuated by increasing antibiotic resistance rates, TPB seems to be the favored approach to address these current challenges. The absence of sepsis after TPB with antibiotic prophylaxis has been shown in single-center series [2, 13], and also when prophylaxis was omitted altogether [14–16].

In this study, we present the results of a retrospective multicenter cohort to evaluate the incidence of infectious and non-infectious complications after TPB without peri-interventional antibiotic prophylaxis. Second, we examined whether the number of biopsy cores was predictive for the occurrence of any complication. Third, we investigated the influence of TPB on erectile function. In our study comprising data from three different urological centers, there was not a single case of sepsis reported. This is consistent with earlier studies investigating the safety of an antibiotics-free TPB procedure [14–18], which underlines the safety advantage of TPB without antibiotic prophylaxis and supports the ongoing initiative to abandon TRB of the prostatic gland [19, 20]. Twenty patients (3.6%) developed infectious complications with a need for antibiotic therapy. This incidence is slightly higher compared to other studies. Recently published data from a Chinese single-center cohort of 2192 patients who underwent TPB without antibiotic prophylaxis showed an infection rate of 1.9% [14]. Two small single-center cohorts with low patient numbers of 177 and 130, respectively, even reported zero infectious complications [15, 16]. The slightly higher infection rate found in our study might originate from our best practice data collection method of following patients with a structured phone interview. This might lead to a more accurate estimation of complication rates. Despite the incidence of infectious complications in the study’s cohort being much lower than infection rates after TRB, it is not negligible and, therefore, it is necessary to identify patients at risk that might benefit from peri-interventional antibiotic prophylaxis. A recent retrospective single-center study identified diabetes mellitus and a history of urinary retention as independent risk factors [14]. Multicenter studies with larger cohorts are warranted to identify patients at risk of developing infectious complications. A higher number of cores may lead to more complications by increasing the likelihood of injuring anatomical structures of the prostate and consecutively leading to AUR and bleeding. Furthermore, obtaining more cores could increment the probability of inoculating bacteria that may result in more infectious complications. In multivariate analyses, we found a significant association between higher core numbers and the occurrence of overall complications, specifically bleeding complications and AUR rates. The correlation of core number and complications was reported in a cohort of 3000 patients who underwent TPB with different biopsy schemes. There were significantly more complications in the groups with 18 or 24 cores compared to 12 cores; however, the type of complications was not further specified [21]. To the best of our knowledge, the specific predictive character of core number for bleeding complications has not been examined before. The finding of no association between the number of cores and the occurrence of infectious complications is consistent with a meta-analysis of 1290 patients that showed that increasing the number of cores did not increase infectious complications [1]. Hence, the transperineal approach offers the possibility to safely increase the number of biopsy cores to improve diagnostic accuracy without increasing the risk of infectious complications. The higher rates of bleeding complications may be tolerable as none of the reported cases exceeded CD Grade 1 [11]. Regarding the influence of the number of cores on AUR rates, it is advisable to identify patients at risk for developing AUR. If extensive biopsy schemes are required, this subgroup may potentially benefit from short-term prophylactic alpha-1 inhibitors.

As a subsidiary result of our study, we want to discuss the 14.4% rate of cases with erectile dysfunction after TPB. Importantly, 49/50 patients were diagnosed with PCa. This suggests the possibility of a psychological cause for erectile dysfunction more than a physiological one. We admit that these results might be influenced by the incomplete response rate in this item. However, our finding is in accordance with an earlier study that showed an association between PCa diagnosis and erectile dysfunction in 85 men who underwent TRB. Subgroup analysis showed a significantly greater change in the post-biopsy International Index of Erectile Function (IIEF)-Score in men with PCa compared to men without [22]. These results highlight the psychological influence on erectile function and potential quality of life impairment as a consequence of their diagnosis in PCa patients that must be kept in mind when treating these. To our knowledge, this is the first multicenter trial to investigate the safety of an antibiotics-free TPB procedure of the prostatic gland. The study’s main strength is the high quality and completeness of our follow-up data as they were collected by structured phone interviews. This allows a more accurate evaluation of complications following discharge compared to solely reviewing patients’ electronic medical records. Furthermore, the inclusion of data from different urological facilities such as one University Hospital, one municipal hospital, and two urological private practices makes our results more generalizable. There are several limitations to the current study that must be acknowledged. Our results are based on a retrospective cohort with slightly varying acquisition periods between the participating centers and there was no comparison group. Second, biopsy systems used for TPB were different among the participating centers and may have influenced our analyses. Third, data collection by structured phone interviews is inferior to standardized questionnaires and non-comparable to studies using standardized questionnaires. This applies in particular to our data concerning erectile function that was only evaluated as a binary outcome in our study and should be assessed pre and post biopsy with validated patient-reported outcome measures such as the IIEF-5 or 15 questionnaires. Fourth, we reported complications according to CD, which is not specific to urology or prostate biopsy.

Further research in larger prospective multicenter cohorts is needed to provide high-level evidence for the safety of an antibiotic-free approach. There is an ongoing randomized multicenter trial comparing the rates of infectious complications after TPB in patients with and without antibiotic prophylaxis (NCT04146142). Preliminary results are expected by the end of 2021.

## Conclusion

TPB without antibiotic prophylaxis is a safe procedure. We found no case of sepsis in our multicenter cohort of 550 patients. This underlines the safety advantage of TPB even without antibiotic prophylaxis and supports the ongoing initiative to abandon TRB of the prostate. A higher number of cores were associated with an increase in overall complications specifically bleeding complications, but not with infectious complications. Post-biopsy erectile dysfunction was mainly present in patients diagnosed with PCa.
